# Role of lysophosphatidic acid and its receptors in health and disease: novel therapeutic strategies

**DOI:** 10.1038/s41392-020-00367-5

**Published:** 2021-02-01

**Authors:** Luiz Henrique Medeiros Geraldo, Tânia Cristina Leite de Sampaio Spohr, Rackele Ferreira do Amaral, Anna Carolina Carvalho da Fonseca, Celina Garcia, Fabio de Almeida Mendes, Catarina Freitas, Marcos Fabio dosSantos, Flavia Regina Souza Lima

**Affiliations:** 1 Instituto de Ciências Biomédicas, Universidade Federal do Rio de Janeiro, Rio de Janeiro, Brazil; 2 Université de Paris, PARCC, INSERM, F-75015 Paris, France; 3 Instituto Estadual do Cérebro Paulo Niemeyer—Secretaria de Estado de Saúde, Rio de Janeiro, Brazil; 4 Instituto de Saúde de Nova Friburgo, Universidade Federal Fluminense, 28625-650 Nova Friburgo, Brazil

**Keywords:** Cell biology, Neuroscience, Biomarkers, Biochemistry, Diseases

## Abstract

Lysophosphatidic acid (LPA) is an abundant bioactive phospholipid, with multiple functions both in development and in pathological conditions. Here, we review the literature about the differential signaling of LPA through its specific receptors, which makes this lipid a versatile signaling molecule. This differential signaling is important for understanding how this molecule can have such diverse effects during central nervous system development and angiogenesis; and also, how it can act as a powerful mediator of pathological conditions, such as neuropathic pain, neurodegenerative diseases, and cancer progression. Ultimately, we review the preclinical and clinical uses of Autotaxin, LPA, and its receptors as therapeutic targets, approaching the most recent data of promising molecules modulating both LPA production and signaling. This review aims to summarize the most update knowledge about the mechanisms of LPA production and signaling in order to understand its biological functions in the central nervous system both in health and disease.

## Introduction

### Lysophosphatidic acid biosynthesis and degradation

Lysophosphatidic acid (1- or 2-acyl-sn-glycerol 3-phosphate/radyl-glycerol-phosphate, LPA) is a bioactive phospholipid that is produced during the synthesis of cell membranes and is described as a robust extracellular signaling molecule present in all eukaryotic tissues and blood plasma.^1,2^ LPA is the smallest bioactive lipid which exerts potent extracellular signaling through its interaction with its six specific G protein-coupled receptors (GPCRs), mediating important responses, such as cell proliferation, migration, and cytoskeletal reorganization.^3–5^ LPA molecules are characterized by a glycerol backbone with the addition of a phosphate group at the sn-3 position and a hydroxyl group and a fatty acid chain in either sn-1 or sn-2 positions, with their variations being due to the different fatty acid chains. LPA can be present both intra- and extracellularly with different production pathways in each one of these contexts.^6^

There is one major pathway involved in LPA extracellular synthesis. In this pathway, membrane phospholipids such as phosphatidylcholine, phosphatidylserine, and phosphatidylethanolamine are the precursors’ molecules to produce lysophosphatidylcholine (LPC), lysophosphatidylserine (LPS), and lysophosphatidylethanolamine, respectively through the action of phospholipase A1 (PLA_1_) or PLA_2_. These lysophospholipids (LPs) are then converted into LPA by autotoxin (ATX), which is mainly responsible for LPA maintenance at a physiological concentration in plasma after birth and also during vascular and embryonic development.^4^ It is well established that ATX is the major source of synthetized LPA, which is derived from membrane phospholipids.^7^ Also, the extracellular production of LPA by ATX is important for their role as bioactive lipids by mediating cellular responses through LPA receptors.^8^

Regarding the intracellular LPA production, at least four pathways are involved in this process: (1) the monoacyglicerol kinase (MAGK) pathway; (2) the phosphatidic acid-phospholipase A1 (PA-PLA_1_) or A2 (PA-PLA_2_); (3) the glycerophosphate acyltransferase (GPAT) synthesis pathway; and (4) the oxidative modification of low-density lipoprotein (LDL) pathway.

**Fig. 1:**
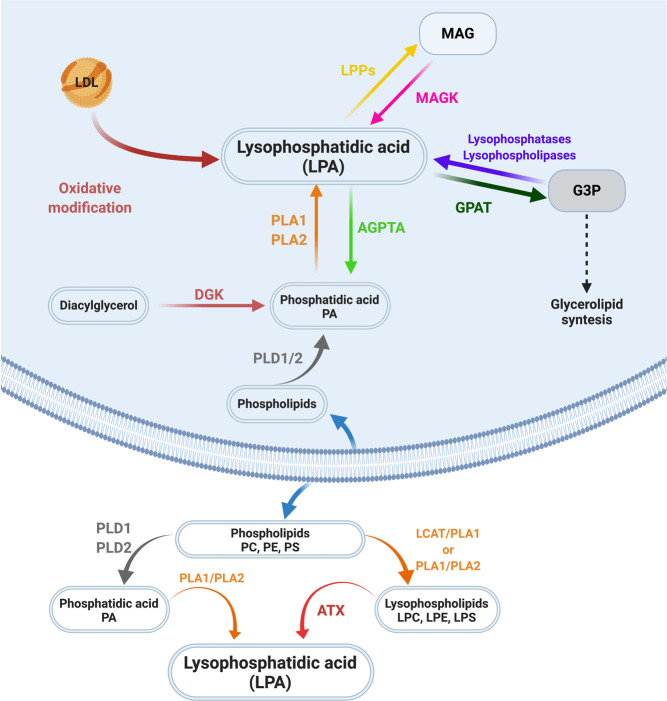
LPA intra- and extracellular biosynthesis and degradation. Several mechanisms are implicated in LPA biosynthesis. Several mechanisms are implicated in LPA biosynthesis. Extracellularly, LPA can be produced by two different mechanisms from phospholipids (ATX dependent or independent mechanisms), and signals through six different transmembrane G protein-coupled receptors. Intracellularly, at least four pathways are involved in this process: (1) the monoacyglicerol kinase (MAGK) pathway; (2) the phosphatidic acid-phospholipase A1 (PA-PLA1) or A2 (PA-PLA2); (3) the glycerophosphate acyltransferase (GPAT) synthesis pathway; and (4) the oxidative modification of low-density lipoprotein (LDL) pathway. Finally, these pathways of intracellular LPA synthesis are the same which leads to its degration. Scheme created using Biorender.com

The MAGK pathway recycles the monoacylglycerol (MAG) produced by the action of lipid phosphate phosphatases (LPPs), allowing the turnover of already catalyzed LPA for another round of signaling.^9,10^ The second pathway involves the production of phosphatidic acid (PA) from phospholipids due to the activity of phospholipase D (PLD_1_ and PLD_2_) or from diacylglycerol by diacylglycerol kinase. Afterwards, PA is converted into LPA through the action of PLA_1_ or PLA_2_ (Fig. 1).^4,11^ The difference between these enzymes is that PLA_1_ removes 1-acyl and thus produces 2-acyl-LPA, whereas PLA_2_ removes 2-acyl and thus produces 1-acyl-LPA. Tissue or cell specific expression of these enzymes can be important for signaling transduction when considering that different LPA receptors can have different binding to 1- or 2-acyl LPA as, for example, LPAR3 and LPAR6 prefer 2-acyl LPA.^12–14^

Also, LPA is an intermediate in glycerolipid synthesis, being produced by GPAT-mediated conversion of glycerol-3-phosphate (G3P). This production of LPA has probably no signaling importance, as this LPA is rapidly converted into PA by acylglycerophosphate acyltransferase (AGPTA, or lysophosphatidic acid acyltransferase) in microssomes (Fig. 1).^15–18^

Finally, all three intracellular mechanisms of LPA production can also be important for its degradation: (1) generation of MAG by LPPs; (2) conversion into PA by AGPTA; and (3) G3P production by lysophosphatases/ lysophospholipases. As we can notice, the intracellular pathways and either produce or degrade LPA depending on metabolites production, expression of the intermediaty enzimes and activation of glycerolipid syntesis.^9,10^ Also, it is important to note that most current evidence suggests that intracellular LPA synthesis and degradation serves as a source of precursors for glycerolipid synthesis and not extracellular signaling molecules.^17,19^

The most common LPA form known in research is described as 18:1 oleoyl-LPA (1-acyl-2-hydroxy-sn-glycero-3-phosphate). There are different LPA species depending on the acyl chain length and so their molecular mass can vary between 430 and 480 Da. Nonetheless, there are many different isoforms of LPA known in biological organisms depending on its phospholipid precursor.^4,20,21^ So, nowadays, there are two different LPA species known, saturated one’s fatty acids (16:0, 18:0) and unsaturated one’s fatty acids (16:1, 18:1, 18:2, 20:4) and, all these LPA species performs distinct biological activities.^22–27^ Moreover, as being so different those different LPA types are recognized by different LPA receptors.^27^

LPA is present in all eukaryotic tissues and fluids under both physiological and pathophysiological conditions. For instance, it has been found in the cerebrospinal fluid (CSF), neural tissue, seminal fluid, saliva, urine, and aqueous humor.^4^ Some studies have also reported the presence of LPA in conditions of nerve injury, in the serum of systemic sclerosis (SSc), in sepsis, in ascites related to pancreatic cancer and in the plasma of patients with chronic liver injury and obesity.^4,5^ LPA concentration significantly differs in the plasma and the serum. While the concentration of LPA in the plasma ranges from 0.7 to 80 nM, in serum it can reach 10 µM.^4,21,28^

### Lysophosphatidic acid signaling and receptors

**Fig. 2:**
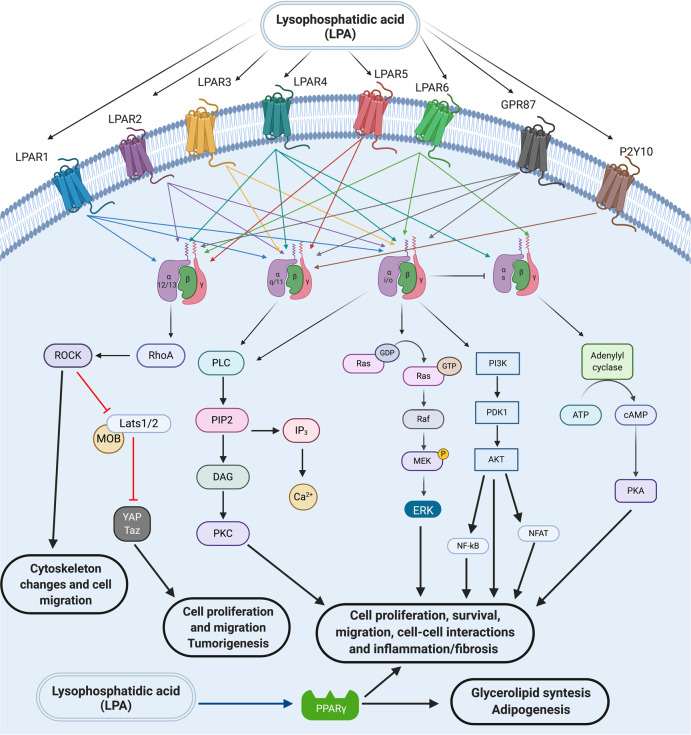
LPA receptors and signaling intermediates. LPA signals through six different LPA transmembrane G protein-coupled receptors (GPCRs and LPAR1–6) and other GPCRs (such as P2Y10 and GRP87). These receptors activate different intracellular signaling mediators to elicit different/several cellular responses (such as cytoskeleton reorganization, migration, proliferation, survival, and cell–cell communication). Finally, LPA was also recently described as a direct PPARγ agonist and in this sense intracellular LPA could also induce signaling through this receptor. Scheme created using Biorender.com

The first evidence for a role of LPA as a bioactive molecule dates from the 1960s with the identification of its effects on blood pressure, platelet activation and intracellular calcium release during smooth muscle contraction.^3,29,30^ Nearly two decades later, in the mid-1980s, a variety of cellular mechanisms, including cell growth, cell morphology, neurite retraction, and the development of actin stress fibers had already been associated with LPA signaling.^31^ However, the molecular mechanistic description of the involved metabotropic receptor with different G protein responses was only elucidated after 1996, when the first LPA receptor was cloned.^32^ Years before, the endothelial differentiating gene (Edg) family had been described. Nevertheless, their ligands had not been identified yet. The first result correlating LPA signaling through these GPCRs showed that the upregulation of Edg2 in murine neuronal cultures could lead to morphological changes and adenylyl cyclase inhibition in response to LPA.^4^ Even before the year 2000, another eight genes encoding Edg receptors in the human genome were identified. Among them, three encoding LPA receptors, namely LPAR1/Edg2,^32^ LPAR2/Edg4^33^ and LPAR3/Edg7,^12,34^ which, in turn, are part of the rhodopsin GPCR family alpha subclass.^35^ In 2003, beyond the classical Edg family LPA receptors, the first non-Edg LPA receptor, which was further called LPAR4, was identified during experiments to find a P2Y9/GPR23 ligand.^36,37^ This discovery opened up the possibility of investigating the existence of other LPA receptors and revealed the existence of two more LPA receptors, the LPAR5 (GPR92/93) and LPAR6 (P2Y5). Together, these three receptors constitute a new family of non-Edg LPA receptors. Such receptors are part of the rhodopsin delta subclass and have stronger homology to P2Y purine receptors (P2YRs) (see Tables 1, 2, and Fig. 2).^3–5,35,38,39^

**Table 1:** LPA receptors: expression, functions, and intracellular signaling mediators

Receptor	G protein	Action	Expression/observations	References
LPAR1 or Edg2	G_α12/13_, G_αq/11_ and G_αi_	Cellular proliferation, serum response elements, MAPK, PLC, Akt, and Rho activation, adenylyl cyclase inhibition, Ca^2+^ mobilization, and YAP/Taz activation	First receptor identified for LPA. Its expression is in the uterus, brain, testis, small intestine, lung, heart, kidney, stomach, spleen, placenta, thymus, and skeletal muscle (adult human and mice). Its expression is more restricted during embryonic development, including portions of the brain, limb buds, dorsal olfactory bulb, somites, craniofacial region, and genital tubercle.	^32^
LPAR2 or Edg4	G_α12/13_, G_αq/11_ and G_αi_	Cellular proliferation, serum response elements, MAPK, PLC, Akt, and Rho activation, adenylyl cyclase inhibition, and Ca^2+^ mobilization	Shows 55% amino acid similarity to LPAR1. During development its expression is at limb buds, brain, Rathke’s pouch, and craniofacial regions. This receptor is high expressed in testis and leukocytes (human and mouse); and kidney, testis, and uterus (mouse). More moderate levels of this receptor are found in the spleen, prostate, pancreas and thymus (human), and lower levels are found in the stomach, lung, spleen, postnatal brain, thymus, and heart (mouse).	^33^
LPAR3 or Egd7	G_αq/11_ and G_αi_	MAPK and PKC activation, Ca^2+^ mobilization, adenylyl cyclase inhibition, and YAP/Taz activation	During development its expression is observed in the mesonephros, heart, and in three spots in the otic vesicle (mouse). In the adult, its expression is most prominent in the testis, heart, pancreas, and prostate (human and mouse) as well as kidney, lung, testis, and uterus (mouse). It is less highly expressed in the ovary, lung and brain (human); and brain, small intestine, heart, placenta, thymus, stomach, and spleen (mouse).	^12,34,35^
LPAR4 or P2Y9 or GPR23	G_α12/13_, G_αq/11,_ G_αi_ and G_αs_	Cellular proliferation, serum response elements, MAPK, PLC, Akt, and Rho activation, adenylyl cyclase inhibition, and Ca^2+^ mobilization	During development, it is found in the embryonic brain, branchial arches, maxillary processes, liver, limb buds, and somites (mouse). In adults, it is prominently found in the ovary, while less prominent in the pancreas, thymus, brain, small intestine, heart, testis, colon, prostate, and spleen (human). It is present in ovary, heart, thymus, skin, and bone marrow (mouse).	^36^
LPAR5 or GPR92/93	G_α12/13_ and G_αq/11,_	Ca^2+^ mobilization and PLC activation	*LPAR5* shares 35% homology with *LPAR4*. It is highly expressed in spleen, and to a lesser degree in placenta, heart, colon, small intestine, and liver (human). It is highly expressed in small intestine and more moderately in heart, lung, stomach, spleen, colon, skin, thymus, platelets, liver, gastrointestinal lymphocytes, mast cells, and dorsal root ganglia (mouse). During development, it was found in the early embryonic mouse rostral midbrain, forebrain, and hindbrain (mouse).	^3–5,35,38,39^
LPAR6- P2Y5	G_α12/13_, G_αq/11_ G_αi_ and G_αs_	Rho activation	Little is known about this receptor, however, genetic studies indicated roles for LPA6 in hypotrichosis simplex, a complex of diseases involving familial hair loss.	^3–5,35,38,39^
GPR87	G_α12/13,_ G_αq/11_ and G_αi_	Intracellular Ca^2+^ increase	It is expressed at high levels in testis and brain and in other tissues, such as placenta, ovary, prostate, and skeletal muscle in a lower level, but it is not expressed in heart, lung, kidney, liver, or intestine	^64^
P2Y10	G_αq/11_	Intracellular Ca^2+^ increase	It is expressed at high levels in appendix, lymph node; and spleen; more moderately in bone marrow, colon, duodenum, reproductive organs, brain, lung, and skeletal muscle.	^69^
TRPV1	Ca^+2^ channel		It is expressed at high levels in duodenum, ovary, skin, small intestine; more moderately in adrenal, appendix, brain, colon, endometrium, kidney, liver, placenta, prostate, spleen, stomach, gall bladder, fat, testis, thyroid, and urinary bladder.	^70,71^

**Table 2:** LPA receptors: molecular characteristics

Receptor	Number of residues	Molecular weight (kDa)	Chromosomal localization of genes in humans	Number of exons	Similarity to other receptors (in %)	Role of various residues in function (interaction with LPA)
LPAR1 or Edg2	364 Amino acids	∼41	9q31.3	Five exons		
LPAR2 or Edg4	348 Amino acids	∼39	19p12	Five exons	∼50% identical at the amino acid level to LPAR1.	
LPAR3 or Egd7	353 Amino acids	∼40	1p22.3–p31.1	Five exons	∼50% identical in amino acid sequence to LPAR1 and LPAR2 (mice)	Preference for 2-acyl-LPA rather than 1-acyl-LPA
LPAR4 or P2Y9 or GPR23	370 Amino acids	∼42	Xq21.1	Five exons	Less than 20% amino acid sequence identity with LPAR1, LPAR2 and LPAR3	
LPAR5 or GPR92/93	372 Amino acids	∼41	12p13.31	Three exons	∼35% homology with LPAR4	
LPAR6- P2Y5	344 Amino acids	∼39	13q14	Ten exons		Preference for 2-acyl-LPA rather than 1-acyl-LPA
GPR87	358 Amino acids	∼41	3q25.1	Three exons	Share 27% and 25% similarity with LPAR4 and LPAR5, respectively	
P2Y10	364 Amino acids	∼39	Xq21.1	Five exons		
TRPV1	839 Amino acids	~100.7	17p13.2	Nineteen exons		Activated only by LPA (18:1)

LPAR1 and LPAR2 signals through G_αi_, G_αq_, and G_α12/13_, which trigger several downstream signaling cascades through PLC, mitogen-activated protein kinases (MAPK), protein kinase B (PKB/Akt) and rho-kinase pathways when LPAR1 is activated; and Ras GTPases, Rac GTPase, phosphatidylinositol 3-kinase (PI3K), MAPK, phospholipase C gamma (PLCγ), diacylglycerol, and Rho in the case of LPAR2 activation. Interestingly, LPAR3 couples only with G_αi_ and G_αq_ to induce calcium mobilization, adenylyl cyclase inhibition and activation, PLC activation and MAPK activation.^4^ Due to these distinct downstream pathways that can be activated, LPA can, therefore, induce a wide range of different cellular responses.

LPAR1 is widely expressed in several organs, including brain, uterus, testis, lung, small intestine, heart, stomach, kidney, spleen, thymus, placenta, and skeletal muscle; and its activation by LPA promotes cell proliferation and survival, cell–cell contact, cell migration, cytoskeletal changes, Ca^2+^ mobilization, and adenylyl cyclase inhibition.^4,31,40,41^ Moreover, recently it was demonstrated that LPAR1 can be considered a novel neural stem/progenitor cells (NSPC) marker as it has an interesting expression pattern in the adult dentate gyrus.^42^ LPAR2 signaling is also involved in cell survival and migration, immune function, myelination, and, like LPAR1, LPAR2 contributes to several aspects of nervous system development, function, and injuries.^4,43^ LPAR3 mediates Ca^2+^ mobilization, adenylyl cyclase signaling, MAPK activation, and presents strong expression in human heart, testis, prostate, pancreas, while it is less expressed in human lung, brain, heart, stomach, placenta, spleen, and thymus.^4,5,40^

In 2012, a new intracellular pathway activated by LPA was dissected. In an attempt to find the components of fetal bovine serum capable of inducing the nuclear localization of yes-associated protein (YAP), the effector of the Hippo pathway, the results pointed out that these components were bioactive lipids.^44,45^ The authors demonstrated that both sphingosine-1-phosphate (S1P) and LPA are capable of promoting YAP dephosphorylation in Ser127 which leads to YAP translocation to the nucleus, inducing the expression of several target genes of the Hippo pathway such as Cyr61 and connective-tissue growth factor (TGF). LPA was able to induce inactivation of large tumor suppressor kinase 1 (Lats 1) activity, an upstream component of the hippo pathway that marks YAP for degradation, while another upstream component, MST 1 showed no change of its kinase activity after treatment with LPA. Using specific chemical inhibitors or silencing RNAs, it was concluded that the actions of S1P occurs through its binding with the S1P2 receptor and not with S1PR1 or S1PR3 while the action of LPA occurs preferably through LPAR1 and LPAR3 that lead to activation of Rho GTPases inducing cell proliferation or cell migration.^44,46,47^ Interestingly, LPA is also able to bind to an intracellular receptor, regardless of its interaction with its receptors LPAR1–6. McIntyre and colleagues demonstrated that LPA is able to bind directly to the Peroxisome proliferator-activated receptor-gama (PPARγ) transcription factor and activates expression of a PPAR-responsive element reporter.^48^

Several interesting works pointed to the involvement of LPA receptors in cell motility. Different actions of LPAR1, LPAR2, and LPAR3 have been demonstrated, for example, in endothelial cells during chemotherapy using cisplatin or doxorubicin. One study reported that chemotherapy itself inhibited the expression of those LPA receptors. In addition, the motility was lower in the treated than in the untreated cells. Noteworthy, such effect was abolished when pancreatic cancer cells (PANC-1) were cocultured with treated endothelial cells, therefore suggesting that this effect was due to the expression of LPA receptors in endothelial cells.^49^ This work aimed to assess the LPA receptors’ roles in cellular responses during chemotherapy and to identify molecular targets involved in proceeding an effective antiangiogenic treatment in combination with chemotherapy.^49^ Surprisingly, it has been found that LPAR1 knockdown on endothelial cells increases cell mobility, while knocking down LPAR2 and LPAR3 inhibits it, suggesting that LPAR1 negatively regulates cell mobility and LPAR2 and LPAR3 positively regulate it in endothelial cells.^49^ This result demonstrated that LPA signaling is an important mechanism involved in modulating cell migration not only during development but also during pathological conditions and in the responses to treatment. Hence LPA signaling might be considered a promising target for cancer treatment, further studies still being necessary to confirm this hypothesis.

LPAR4, the first receptor described with similarity to P2Y purinergic receptors, can couple with the different G protein, G_αs_, G_αi_, G_αq_, and G_α12/13_.^4,39,50,51^ This receptor is strongly expressed in the human ovary, while it is less expressed in other organs, such as thymus, pancreas, colon, brain, heart, small intestine, testis, prostate, and spleen.^4^ Interestingly, LPA-induced cell migration through LPAR1 is inhibited when LPAR4 is activated, which shows a negative regulation loop of cell mobility induced by LPA. LPAR4-deficient mouse embryonic fibroblasts showed higher response to LPA-induced cell migration and reconstitution of LPAR4 on LPA-negative cells showed a less motile phenotype.^37^ Moreover, in B103 neuroblastoma cells without endogenous LPA receptors, the co-expression of ectopic LPAR4 with LPAR1 impaired LPAR1-driven migration and invasion.^37^ Another study also demonstrated that ectopic expression of LPAR4 in SQ-20B cells, a head and squamous cell carcinoma, expressing normal level of endogenous LPAR4, abolished LPA-induced motility as well as by Ki16425, Rac1, or Y-27632 LPA receptors inhibitors.^52^ More recent, it was shown that the LPAR4 knockdown in human pancreatic PANC-1 cells, PANC-sh4, the cell motility were enhanced accompanied by markedly stimulation on invasive activities.^53^ The same effect occurred on LPAR5 receptor knockdown PANC-1 cells, but not on LPAR6 receptor knockdown PANC-1 cells, which shows the distinct roles of LPA receptors in pancreatic cancer cells.^53^ In addition to other well-described functions of LPAR4 activation, such as cell aggregation, cell adhesion,^54^ vascular development,^55,56^ and osteogenesis regulation,^57^ it has recently been demonstrated the molecular mechanism of hypertensive response mediated by LPA.^58^ Different LPA analogs acted as LPAR agonizts, leading to hypertensive responses, and pretreatment with a Rho-kinase inhibitor that blocked G_α12/13_ signaling attenuated this hypertensive effect induced by LPA. Corroborating this finding, another study reported that this effect is decreased in LPAR4 knockout mice, a G_α12/13_-coupling LPA receptor.^58^ That study suggested a correlation between circulating LPA, produced by ATX, and the increase in blood pressure through its receptors, particularly LPAR4.

On the other hand, LPAR5 binds to G_αq_ and G_α12/13_, G_αi_ and G_αs_.^3,4,39,59^ LPAR5, identified in 2006, is highly expressed in the spleen and less expressed in the heart, small intestine, placenta, colon, and liver.^4^ This LPA receptor also negatively regulates cell motility as LPAR4 and is implicated in the induction of chemokine release.^3^ Moreover, LPAR5 is also responsible for LPA-induced neurite retraction and cytoskeleton stress fiber formation through a signaling cascade downstream of G_α12/13_.^60^

LPAR6 binds to G_α12/13_ and is the most recently discovered LPA receptor. Mutations in this receptor have been identified in patients with autosomal recessive hypotrichosis. Such findings indicate that LPAR6 may be a potential therapeutic target for human autosomal recessive hypotrichosis (alopecia).^61^ LPAR6 continues to be investigated to better understand its functions.^3^ Interestingly, LPAR6 was also related with prostate cancer progression in a past work.^62^ Recently, the crystal structure of LPAR6 was determined with the purpose of elucidating the ligand recognition mechanism of the non-Edg family of LPA receptors. This study was important for the identification of LPAR6 antagonists, which might provide with new therapeutic drugs for cancer treatment.^63^

GPR87 is also known as a LPA receptor since it was deorphanized in 2007 by Tabata and collaborators. Using CHO cells transfected with pcDNA5 vector containing GPR87-G_α16_ fusion gene, they examined the effects of P2Y and P2Y-related receptor ligands on intracellular Ca^2+^ ([Ca^2+^]_*i*_) movements of GPR87 cells and showed that LPA significantly stimulated a [Ca^2+^]_*i*_ increase in a dose-dependent manner. They also demonstrated that this effect was not related with the endogenous LPA receptors comparing LPA effects on the wild type and GPR87-G_16α_ expressing cells. As a result, the GPR87 cells showed a higher Ca^2+^ response to LPA than the wild type cells. Besides that, different P2Y and P2Y-related receptor agonist such as ATP, UDP-glucose, phosphoribosyl pyrophosphate, α-ketoglutarate, leukotrienes, and phospholipids were analyzed to intend its capacity on activate GPR87 but only LPA significantly increased GPR87-mediated [Ca^2+^]_*i*_ levels.^64^ Furthermore, they used siRNA to knockdown the expression of GPR87 which completely inhibited the LPA-induced Ca^2+^ response. The amino acids sequence homology of GPR87 with LPAR1, LPAR2, and LPAR3 is insignificant while share 27 and 25% similarity with LPAR4 and LPAR5, respectively. By the other hand GPR87 showed 41–48% amino acid identity with P2Y12, P2Y13, and P2Y14 receptors.^64^ The GPR87 gene is expressed at high levels in testis and brain and in other tissues, such as placenta, ovary, prostate, and skeletal muscle in a lower level, but it’s not expressed in heart, lung, kidney, liver or intestine.^64^

In 2013, a study addressed the role of GPR87 in urothelial carcinoma of the bladder through its ligand LPA. They showed a substantial expression of GPR87 gene in five human bladder cancer cells lines (HT1197, J82, TT112, RT4, and TCCSUP) and its correlation with cellular viability following gene silencing experiment. After GPR87 mRNA level suppression with transfection of Ad-shGPR87 until 120 h, they observed a significant reduction on cellular viability in a dose-dependent manner. Moreover, 38 of 71 non-muscle-invasive bladder cancers (54%) was positive for GPR87 immunostaining, were the positive-staining ratio in high-grade tumors showed a tendency to be higher than that in lower-grade tumors. GPR87-positive tumors also showed a higher median Ki-67 index than that in GRP87-negative tumors, thus a strong correlation with cell proliferation.^65^ As they have also reported enhanced expression of ATX in prostate cancers correlated with tumor grade and capsular invasion, they suggest that GPR87 is a potential prognostic marker for the progression of non-muscle-invasive bladder cancers.^65,66^ Also, in 2013, the same research group used A431 cells, a human epidermoid cancer cell, which previously have been shown to express GPR87, to study whether GPR87 acted as an LPA receptor.^67^ They demonstrated that when the expression of GPR87 was decreased by treatment with *gpr87*-specific siRNA, LPA-induced colony dispersal was significantly diminished. Before, Kam and Quaranta showed that A431 cells treated with LPA rapidly and synchronically dissociate cadherin-based adherents junctions^68^. Thus, they predicted that the LPA-induced cell scattering was via activation of GPR87 in A431 cells. The signaling pathways underlying this process were addressed and Erk, JNK, p38, and Akt were significantly activated 10 min after addition of LPA. Further, A431 cells were pretreated with the signaling pathways inhibitors, LPA-induced A431 colony dispersal was blocked completely.^68^

Another orphan GPCR, P2Y10 receptor was identified as LPA and S1P receptor in 2008.^69^ LPA was capable to evoke intracellular Ca^2+^ increase in the CHO cells which were inhibited by LPA receptor antagonists. In order to corroborate with these findings, they performed an assay using a siRNA designed for P2Y10 receptor into the P2Y10-CHO cells and showed that LPA-induced Ca^2+^ increased was blocked. Reverse transcription polymerase chain reaction (PCR) analysis showed assed its expression in different mice tissues, as reproductive organs, brain, lung, and skeletal muscle suggesting physiological roles.^69^

The transient receptor potential vanilloid 1 (TRPV1) ion channel responds to various stimuli, including LPA, which is also associated with neuropathic pain. Structural affinity determines the activation of TRPV1 and among the natural LPA analogs, Morales-Lázaro et al. found that only LPA 18:1, alkylglycerophosphate 18:1, and cyclic PA 18:1, all with a monounsaturated C18 hydrocarbon chain, are capable to activate TRPV1.^70^ The involvement of LPA in pain was first demonstrated in bone cancer pain through to its capacity to potentiate TRPV1 in dorsal root ganglion (DRG) neurons.^71^ Immunohistochemistry staining showed high levels of co-localization of LPAR1 with TRPV1 in DRG neurons and TRPV1 current were potentiated by LPA in isolated DRG neurons.^71^ Recently, a study aimed to underlie the mechanism by which LPA activates peripheral sensory neurons involved in intractable and continuous itch sensations present in atopic dermatitis, neurogenic lesions, uremia, and cholestasis, since LPA is an itch mediator found in cholestatic itch patients.^72^ They used a cheek injection model and the experiments revealed that the LPA-induced itch was dependent of transient receptor potential ankyrin 1 (TRPA1) and TRPV1, which leads to conclude that TRPV1 is one of the potential targets for cholestatic itch future treatments.^73^

## LPA and CNS development

LPA is undoubtedly very important during embryonic development, as this bioactive lipid signaling regulates processes, such as cell proliferation, survival, differentiation, adhesion, migration, and morphology.^19,74–80^ The concentration of LP was found to vary greatly from nanomolar to micromolar ranges among different embryonic tissues, such as the brain, choroid plexus, neural tube, blood vessels, meninges, CSF, and spinal cord.^81,82^ Acting through specific GPCRs in a paracrine or autocrine fashion, LPA influences the formation and maintenance of the central nervous system (CNS). It was already demonstrated that LPA influences neuron–glia interaction, driving events such as neural progenitor cells (NPCs) commitment and maturation, and oligodendrocyte differentiation.^76–78,83^ Moreover, it was also demonstrated that LPA within certain lower spectrum of concentrations may function as an extracellular signal inducing the proliferation of NSPCs and their differentiation to specific cholinergic-committed neurons and unspecific microtubule-associated protein-2 (MAP2)-positive neurons.^84^ Normal brain development depends on LPA signaling and LPAR modulation and it is well established that LPAR expression occurs in a shifting spatio-temporal arrangement.^5^ To date, six LPARs have been described (LPAR1–6 for human and Lpar1–Lpar6 for nonhuman), however, only Lpar1, Lpar2, Lpar4, Lpar5, and Lpar6 have been detected during brain development.^4,85,86^ In the developing brain Lpar1 and Lpar2 are principally expressed in the ventricular zone (VZ) of the cerebral cortex, which is correlated with initiation, progression, and decline of neurogenesis.^32,87^ Moreover, *Lpar1*-null mice exhibit usually 50% of perinatal lethality that is correlated with defective suckling behavior which may be due to olfactory bulb and cerebral cortex defects, although no obvious abnormalities in the cerebral cortex were observed.^88^ Another variant of *Lpar1*-null mice (called “Málaga variant” or *maLPAR1*-null mice) exhibited a reduced VZ, premature neural progenitor cell maturation, and an increase in cortical apoptosis that causes a defect on the cortical layers in adults.^89^ Recently, it was demonstrated that LPAR1 is also expressed in some distinct areas of the brain, such as the amygdala, where there is a possible crosstalk with the endocannabinoid receptor 1 (CB1), where the lack of CB1 receptor in the amygdala promotes the increase in LPAR1 in this area.^90^ Interestingly, it was demonstrated that exogenous LPA exposure of ex vivo cerebral cortex induces gyri-like folds and widening of the cerebral cortex wall due to an increase in terminal mitosis and a decrease in cell death of the NPCs within the VZ.^75^ Exposure of ex vivo *Lpar1–Lpar2*-null mice cerebral cortex to exogenous LPS did not show these same effects, demonstrating that it was receptor mediated.^75^ LPA/Lpar1 signaling was also demonstrated to be important for survival and development of dopaminergic neurons (DA).^91^ Furthermore, the absence of Lpar1 signaling reduces not only cortical neuronal development but also cortical oligodendrocyte function and myelination.^83,89^

When ATX, (ENPP2), major enzyme responsible for extracellular LPA production from the precursor LPC, is constitutively removed, it results in vascular and neural tube defects with embryonic death around E9.5.^92–94^ In zebrafish, it was demonstrated that *Enpp2* coordinates oligodendrocyte differentiation, showing that the important role of LPA in the developing brain is evolutionarily conserved.^95^ It was also demonstrated that ATX regulates cell positioning and adhesion of neuronal progenitor cells located in the VZ of the cerebral cortex. Moreover, ATX depletion leads to proliferation defects and alterations in the cell cycle.^96^

In situ data have demonstrated that, besides *Lpar1*, *Lpar2*, *Lpar4*, *Lpar5*, and *Lpar6* are also expressed in the early developing brain.^85,97^ *Lpar4* expression was observed mainly in the prospective midbrain–hindbrain boundary and in the margins of the neural folds, from the level of the rostral hindbrain to the forebrain, at E8.5. Later, at E11.5, it is also expressed at the midbrain and hindbrain.^85^ *Lpar5* was strongly expressed at E8.5 in the rostral midbrain, forebrain and margins of the neural folds, and exhibited a diffuse expression in the developing brain including the choroid plexus.^85^ *Lpar6* was observed in the neural plate of *Xenopus neurulae* and its depletion results in forebrain and hindbrain defects.^97^

As cortical neurogenesis occurs in mice from E10 to E18, LPA signaling plays an important role in NPCs’ proper commitment and influences directly young postmitotic neurons.^75,98^ It was shown that LPA can affect in vitro and in vivo neuronal migration by altering the actin cytoskeleton and promoting microtubule rearrangement in neurons.^99,100^ Moreover, LPA also improved the commitment of neuroblasts to the neural lineage through the Lpar1–G_αi_ pathway, as the exposure of *Lpar1–Lpar2*–null neuroblasts to LPA failed to recapitulate the same result.^98^

Neuron–glia interaction plays a pivotal role in brain development and, quite expectedly, LPA is also involved in this interaction. In vitro, LPA-primed astrocytes induced neuronal commitment by activating Lpar1–Lpar2. These events were mediated by modulation of laminin and fibronectin organization by astrocytes. The activation of Lpar1–Lpar2 induced astrocytes to secrete epidermal growth factor ligands (EGF and TGFα) and the activation of the MAPK cascade and protein kinase A (PKA) signaling pathways in response to LPA.^76–78^ LPA also stimulates, via the LPAR1, neuronal differentiation of neural progenitors, cortical neuroblasts, and early cortical neurons.^98^ There is no doubt that LPA is an essential factor for cortical neurogenesis, as it is able to induce depolarization of mouse cortical neuroblasts and to activate electrical responses in neuroblasts via gamma-aminobutyric acid signaling.^101,102^

## LPA and CNS angiogenesis

Brain endothelial cells lie at a strategic position and function as a barrier for the exchange between circulating lipids and the brain parenchyma. Interestingly, an increasing set of in vitro and in vivo data points to a direct effect of the blood-borne bioactive LPA ligand in CNS angiogenesis and endothelial cell permeability, highlighting the importance of LPA signaling in various CNS pathologic conditions where vascular integrity is compromised. During development, after the initial phase of angiogenic sprouting from the perineural vascular plexus, CNS endothelial cells acquire unique features such as expressing specific tight junction proteins and membrane transporters establishing a selective blood–brain barrier (BBB) to regulate ion and molecule transfer to the cerebral microenvironment and ultimately ensure proper neural function.^103^ A more consistent body of literature has addressed the role of LPA signaling in BBB integrity rather than a role on CNS angiogenic sprouting and regulation, although, genetic deletion of ATX results in embryonic lethality due to the severe yolk sac and embryonic vascular malformations,^92^ demonstrating that LPA signaling is essential for overall vascular development, likely through LPA receptor-coupled G protein_α13_.^104^ Data from genetic deletion studies of *Lpar1–2* indicate a mild vascular phenotype, with some incidence of perinatal frontal hematoma,^40,43^ while *Lpar4*-deficient mice display several developmental vascular defects leading to embryo lethality.^55^ In a recent study, Yasuda and collaborators demonstrated the synergistic role of the LPA receptors Lpar4 and Lpar6 during vascular development. *Lpar4*/*Lpar*6 double-knockout (DKO) embryos dye around E11.5, presenting enlarged aortae, reduced vascular tree in the cephalic region, as well as yolk sac vascular defects.^105^ Further dissection of the molecular mechanism shows that LPA through its endothelial receptors Lpar4/Lpar6 represses β-catenin- and Notch intracellular domain-mediated dll4 activation via G_α12_/_α13_-dependent activation of YAP-Taz.^105^

High levels of LPA have been shown to be increased in several models of brain pathologies in which the BBB is disrupted, such as intracerebral hemorrhage, cerebral ischemia, and edema.^106–108^ Also, in glioblastoma (GBM), a highly vascularized brain tumor with associated BBB disruption, ATX is also strongly expressed.^109^ In addition, LPA modulates cerebral circulation, as shown by dose-dependent local application under a cranial window in piglets.^106^ Dissecting the mechanisms of LPA in brain endothelial cell biology and BBB maintenance will be critical to understand its role in angiogenesis-mediated brain diseases as well as a possible therapeutic target both to treat brain diseases and to enhance drug delivery.

LPA receptor mRNA profile in vitro studies determined that *Lpar1–4* are expressed in human and bovine brain microvascular endothelial cells (BMECs)^110^ and *Lpar6* is present in rat BMECs.^108^ While in vivo studies demonstrated that *Lpar1–4* mRNA transcripts were present in whole brain homogenates, robust *Lpar1*, *4*, and *5* mRNA expressions were detected in brain capillary-enriched fractions.^110^ The effects of LPA on BBB permeability were determined through transendothelial electrical resistance and fluorescence transfer across the endothelial monolayer studies which demonstrated that LPA disrupts BBB integrity in different mammalian BMECs, including human.^108,110–112^ LPA induced a disruption in the junctional expression pattern of several BBB characteristic tight junction proteins, such as occludin, zonula occludens-1, and claudin-5 in rat BMECs,^108^ likely due to cytoskeletal rearrangements shown by F-actin stress fiber and focal contacts in LPA-treated porcine BMECs.^111^ Fulminant hepatic failure leading to BBB breakdown and cerebral edema were correlated with a significant increase in ATX serum levels in mice.^108^ The effect of stereotaxic microinjections of LPA in the mouse striatum leads to disruption of BBB as determined by Evans blue dye extravasation and ultrastructural electron microscopy analysis.^113^ Co-injection with the Rho and ROCK (Rho-associated protein kinase) signaling pathway inhibitor Y26732 indicated that LPA-induced BBB breakdown likely occurs through matrix metallopeptidase 9 (MMP9) and urokinase-type plasminogen activator (uPA) proteolytic enzyme activities mediated by the Rho/Rock signaling pathways.^113^ The BBB permeability induced by LPA was further analyzed in mice in terms of onset, duration, and magnitude using magnetic resonance imaging (MRI) and near-infrared fluorescence imaging.^110^ LPA-induced BBB breakdown occurred within minutes as demonstrated by MRI studies in mice using a low molecular weight contrast agent. Twenty minutes after LPA administration, the BBB integrity was restored as a second injection with contrast agent alone did not lead to increased signal, indicating that BBB disruption is a rapid and transient phenomenon. Ex vivo brain slice analysis by near-infrared fluorescence imaging using a large macromolecule contrast agent showed LPA-induced penetration in brain tissue within 20 min. The authors further tested the potential use of LPA to enhance drug delivery to the brain parenchyma. By co-injecting LPA with radiolabeled methotrexate, they showed that radioactivity detection in the brain fraction vs. plasma was increased in LPA-treated animals.^110^ Overall, these studies focused on the role of LPA in BBB integrity have highlighted a direct role of this LP on CNS endothelial cells. Although details of the mechanisms of LPA through G-coupled receptor signaling on non-CNS endothelial cells have been elucidated,^114^ only recently a study has focused on the role of LPA in different steps of CNS angiogenesis.^115^ Analysis of inducible endothelial-specific *Lpar4*/*Lpar6* DKO retinas revealed a clear phenotype on retinal vascular expansion, tip cell, and sprouting morphogenesis. In this study, a series of elegant in vivo and in vitro assays allowed the authors to identify LPA plays an important role in CNS angiogenesis through Lpar4/Lpar6-mediated suppression of the angiogenic regulator dll4 through G_α12_/_α13_-dependent activation of YAP-Taz.^115^ Future studies will be important to evaluate a possible role of these pathways in BBB integrity both in physiological and pathological conditions.

## LPA and neuropathic pain

Pain is considered as an important clinical symptom common to several diseases. Furthermore, it is a warning signal related to the presence of actual or potential tissue injury.^116^ Pain might be classified into acute and chronic, and an additional classification subdivides pain into two other categories (neuropathic and nociceptive pain).^116,117^ Neuropathic pain is defined as “pain that arises as a direct consequence of a lesion or disease that affects the somatosensory system” where a transduction process is no longer needed to trigger neuropathic pain.^118^ Epidemiological studies have estimated that chronic pain affects nearly 100 million adults only in the United States,^119^ and neuropathic pain affects around 7–8% of the general population. Therefore, chronic neuropathic pain produces huge impacts on the quality of life of the affected individuals.^120^ Interestingly, multiple and sequential peripheral and central mechanisms have been proposed to explain the development and maintenance of neuropathic pain.^121^ Among those, LP are considered important players in the pathophysiology underlying the establishment of this type of pain. Of particular interest is LPA, a lipid mediator released under conditions of tissue injury. Both ATX, LPA, and its precursor LPC have been proved to participate in the mechanisms of neuropathic pain.^122^

The synaptic reorganization promoted by LPA via LPAR1 is one of the most discussed mechanisms in neuropathic pathophysiology. This complex process has been characterized by the presence of C-fiber retraction along with A-fiber demyelination and subsequent sprouting of dorsal root fibers, in which both processes would contribute to the development of allodynia, that can be interpreted as the occurrence of pain in response to innocuous stimuli.^122–124^ Although the molecular basis of such synaptic reorganization induced by LPA have not yet been totally clarified, there is evidence that partial sciatic nerve ligation (pSNL), an experimental model of neuropathic pain, promotes demyelination of dorsal root fibers through a pathway that encompasses the activation of RhoA and ROCK.^125^ LPA signaling via G_α12/13_ activates the GTPase RhoA, which is then translocated to the plasma membrane where it activates multiple effectors such as Rho-kinase and ROCK.^126^ In fact, there is also evidence providing a direct link between Rho-ROCK and the development of neuropathic pain.^127–130^ However, that is not the only process associated with LPA-induced synaptic reorganization. Previous studies showed that LPA can induce the silencing of myelin basic protein and its transcription factor Sox10 in cultured S16 Schwann cells,^131^ as well as demyelination and downregulation of myelin proteins in ex vivo cultures of dorsal root fibers.^132^ A further study demonstrated the occurrence of demyelination, downregulation of MAG, which correlates to the development of sprouting of sensory fibers, and damage of Schwann cell partitioning of C-fiber-containing Remak bundles in the dorsal root and sciatic nerve, following sciatic nerve injury.^133,134^ Interestingly, the same changes could not be demonstrated in the dorsal root of LPAR1 receptor-deficient (*Lpar1*^*−/*^^−^) mice. Nevertheless, ex vivo experiments with LPA revealed the presence of demyelination in the sciatic nerve, spinal nerve, and dorsal root. In addition, it was also demonstrated that dorsal root demyelination induced by nerve injury is significantly reduced in mice heterozygous for autotaxin (*Atx*^*+/−*^). Confirming those findings, the addition of LPC to ex vivo dorsal root fibers cultures failed to produce demyelination in the absence of recombinant ATX. However, pronounced demyelination occurred in the presence of ATX. Finally, the intrathecal injection of LPC resulted in potent dorsal root demyelination, which was lessened or even eliminated in *Lpar1*^−/−^ or *Atx*^*+/−*^ mice. Such results suggest that the mechanisms that mediate the conversion of LPC to LPA would be necessary to induce the demyelination associated with neuropathic pain.^122^

Based on the results from some studies, a feed-forward system, which would be responsible for an amplified LPA production following pSNL, has been proposed.^122,124^ According to this model, the production of LPA depends on the simultaneous stimulation with N-methyl-d-aspartate and substance P, causing a synergistic and strong activation of cytosolic and calcium-independent phospholipase A2 (c/iPLA2), which in turn converts phosphatidylcholine to LPC. Afterward, ATX converts LPC into LPA. LPA would then stimulate microglia, acting through LPAR3, leading to increased production and release of interleukin-1β (IL-1β), which would then promote an upregulation/activation of c/iPLA2 in the adjacent neurons. The final result is an amplified production of LPA, associated with peripheral nerve lesions.^135,136^ Such a model would explain the production of LPA following pSNL, in experimental models of neuropathic pain, which nonselectively stimulates different types of peripheral sensory fibers, a process that does not occur in experimental models of inflammatory pain, which preferentially stimulates C-fibers.^122^

It has also been reported that LPA also plays an important role in the early phase of neuropathic pain following peripheral nerve injury, since the LPAR1 antagonist Ki-16425 completely blocked LPA-induced neuropathic pain-like behaviors in a time-dependent manner. For instance, such blockage peaked at 3 h after the peripheral nerve injury. However, it was not maintained after this period. In addition, the administration of Ki-16425 at 3 h but not at 6 h after the peripheral lesion produced a reduction of substance P in the dorsal horn of the spinal cord and an upregulation in the expression of voltage-gated calcium channel alpha(2) delta-1 subunit in the DRG.^137^ Although those results indicate that LPA is mainly related to the early phases of neuropathic pain, it has been largely demonstrated that the production of LPA also stimulates microglia through LPAR3 mechanism, leading to a higher release of IL-1β, activation of c/iPLA2 in the adjacent neurons and consequent amplified LPA production.^135,136^ Moreover, acting through LPAR1, LPA mediates dorsal root demyelination, which has also been related to neuropathic pain-behaviors and LPAR1 signaling upregulates Ephrin B1 and the α2δ1 calcium channel subunit (CA_V_α2δ1) in the DRG.^125,138,139^ Interestingly, gabapentinoids (e.g., pregabalin and gabapentin), one of the most widely used groups of drugs used in neuropathic pain treatment, bind to CA_V_α2δ which makes the connection between LPA and CA_V_α2δ1 extremely useful and attractive for future pharmacological studies.

Distinct roles have been attributed to microglia and astrocytes in the mechanisms of LPA-induced neuropathic pain, being activated astrocytes responsible for the maintenance of neuropathic pain. With this respect, one study showed that the initial pSNL-induced LPA production in the dorsal horn is blocked by depleting microglia or inhibiting its activation (e.g., with minocycline or Mac1-saporin) but not by l-a-aminoadipate (l-AA), gliotoxin that promotes a pharmacological ablation of astrocytes.^140^ Such findings lead some authors to elaborate the hypothesis that the LPA produced through microglial activation might act on astrocytes, inducing the release of chemokines and therefore contributing to the chronic phase instead of the acute neuropathic pain.^122^

Confirming the findings from experimental studies, a recent clinical study evaluated the concentrations of 12 species of lysophosphatidic acids, LPC, ATX, and the phosphorylated neurofilament heavy subunit in the samples of CSF of neuropathic pain patients. Regarding the LPA species, LPA 16:0, 16:1, and 18:1 were correlated with the average pain intensity, while the other LPA species were not. Moreover, the total amount of ATX and LPC was not associated with the pain intensity, suggesting that those molecules would act more mediating the development of neuropathic pain through LPA production than directly inducing neuropathic pain. Noteworthy, the association between LPA 18:1 and the clinical pain (subjectively reported by the studied patients) has been proved to be responsible for LPA amplification through LPAR1/LPAR3 and microglial activation.^141^

In the search for novel therapies to treat neuropathic pain, LPA antagonists emerge as promising candidates. For instance, in a recent work, the novel LPAR5 antagonist AS2717638 proved to have high potency and selectivity and was able to cross the BBB and penetrate the CNS.^142^ The effects of AS2717638 on mechanical allodynia and also in thermal hyperalgesia suggest an important outcome of this LPA antagonist on both TRPV1 and non-TRPV1 expressing neurons. This potentially provides an advantage to duloxetine and pregabalin, two of the most common pharmacological agents used to treat neuropathic pain symptoms, and that preferentially act by suppressing the symptoms related to the activity of non-TRPV1-expressing neurons. In fact, the analgesic effects of the novel LPAR5 antagonist AS2717638 might encompass not only neuropathic but also inflammatory pain.^142^

Studies also linked LPA to the neuropathic component of osteoarthritis (OA).^125^ In addition, a very recent study showed gender differences in several features of LPA-induced joint neuropathy.^143^ For instance, females exhibited more pronounced mechanical allodynia and saphenous nerve demyelination than males following LPA injection. Interestingly, blockade of voltage-gated sodium channel Nav1.8 decreased the allodynia driven by LPA treatment. Such results suggest sex-specific effects on LPA-induced joint neuropathy and a possible role of voltage-gated sodium channels in such mechanisms with possible future application in the development of pharmacological agents to treat OA and more particularly its neuropathic component.^143^

## LPA and neurodegenerative diseases

LPA signaling has been reported to influence synapse formation, synaptic transmission and survival of mature postmitotic neurons,^144,145^ playing an important role in cerebral cortex formation and function.^76–79^ However, the impairment of LPA signaling has also been implicated in several neurological disorders such as schizophrenia, Alzheimer’s, and Parkinson’s disease (PD).^146,147^

PD is a progressive neurodegenerative motor disorder characterized by the selective loss of DA in the substantia nigra, that results in a pronounced depletion of dopamine in the striatum, to which neurons from the substantia nigra send their projections.^148^

In 2014, it was demonstrated, using PD rat models induced with 6-OHDA (6-hydroxy dopamine), that LPA signaling plays a role in the degeneration of DA and in the differentiation of adult rat mesenchymal stem cells (MSCs) into DA neurons. MSCs treated with LPA developed characteristic neuronal morphology and expressed the neuronal marker, neuron-specific enolase, while the expression of glial markers was absent. In this study, although a decrease in the Lpar1 expression in the substantia nigra of 6-OHDA PD rats was reported, there was no significant change in the striatum. The downregulation of Lpar1 expression in the 6-OHDA PD model, along with the differentiation of MSCs into DA neurons following LPA treatment, suggests that LPA/Lpar1 signaling pathway plays a key role in the development and maintenance of DA neurons. In addition, the low expression of Lpar1 in the 6-OHDA PD model may result in the death of DA neurons and thus contribute to the pathogenesis of PD.^91^

In Alzheimer’s disease (AD), the dysfunction in LPA signaling is associated with an impairment in expression and activity of autotaxin.^149^ Both LPA and ATX are highly expressed in the CNS and have been implicated in AD pathogenesis.^150,151^ Decreased levels of antioxidant enzyme activity and increased lipid peroxidation were observed in AD patients in comparison to healthy non-demented control subjects.^152,153^ The increase in lipid peroxidation triggers the neurotoxic events described in AD.^154^ One of the most vulnerable lipids to peroxidation is LDL.^149^ The oxidized LDL, whose LPA is the major bioactive component, disrupts the BBB, contributing to the pathogenesis of AD.^155^ Besides, high levels of oxidized LDL are a key contributor to β amyloid (Aβ) production and deposition in the brain parenchyma, which is the main pathophysiological mechanism in AD.^156^ The Aβ accumulation results in hyperphosphorylation of tau protein, which leads to synaptic failure and consequently, neuronal death.^157^

LPA signaling is present in other diseases that are a prominent risk factor for the development of AD, such as brain trauma.^149^ In traumatic brain injury (TBI), the increase in LPA activity derives from the upregulated expression of LPAR1, LPAR2, and LPAR3.^4^ Autopsy findings, from patients who died after brain trauma, showed diffuse Aβ plaques in the adjacent areas to the lesion site.^158^ The mechanisms responsible for the Aβ accumulation are most likely to be related with the BBB disruption, leading to ischemic insult and increasing the activity of β and γ secretases.^159^ In TBI, the increase of LPA and its metabolites seems to be involved in the early pathologic processes after the trauma, such as neurite retraction, reactive gliosis, inflammation, and cell death.^160^ Using a rat model of neurotrauma, Mcdonald and coworkers demonstrated that LPA increases diffusely after the injury, whereas LPA metabolites increases in white matter, in the hemorrhagic pericontused gray matter regions both LPA and its metabolites were increased, these events results in axonal injury and cell death, especially in the white matter.^161^

In patients, LPA levels increases in the blood and CSF within 24 h in TBI patients, indicating that LPA could possibly be one of the first mediators responding to the injury and suggesting it may be a biomarker for subsequent cellular pathology.^161^

The association between LPA and brain trauma provides indirect evidence that the impairment of LPA signaling can contribute and predispose to the pathogenesis of AD.^162^ In view of these evidences, ATX, LPA, and its receptors become promising therapeutic targets in neurological disorders.

## LPA and cancer

Several studies have linked increased LPA signaling with the biological events related to carcinogenesis and tumor progression, as malignant transformation, increased cancer stem cell proliferation, enhanced invasion and metastasis, reprogramming of tumor and metastatic microenvironments, and development of therapy resistance.^163,164^

ATX and LPARs present numerous roles in tumor cells. Briefly, LPAR1 and LPAR4 have been mostly associated with cell motility and invasion.^37,165–167^ LPAR2 has been connected with cell survival and apoptosis inhibition.^168^ Both LPAR3 and LPAR6 have been implicated in the promotion of tumor proliferation, migration, invasiveness, aggressiveness, and progression of different cancers;^169–172^ besides, LPAR3 has also been related to the regulation of dendritic cell migration, possibly having a role in antitumor immunity,^173^ whereas LPAR5 has been involved with immune response inhibition and cancer progression.^174–177^ ATX and LPA are released by both tumor cells and cells of the tumor microenvironment (TME) increasing invasion, suppressing antitumor response and contributing to therapy resistance of cancers.^176–182^ LPA–LPAR signaling has been demonstrated to promote epithelial–mesenchymal transition (EMT), essential for cancer stem cells invasive and metastatic properties.^183–187^

Another important feature of LPA in TME is the induction of proinflammatory cytokines production, such as IL-6 and IL-8, impacting the progression and metastasis of tumors. Moreover, there is a positive loop in which the inflammatory milieu induces more LPA–LPAR signaling and chronic inflammation can lead to malignant transformation.^188,189^

LPA is a highly studied molecule in cancer. Based on cancers’ importance—regarding high incidence and mortality, investigation prevalence, and our group expertize, we decided to devote this section to discuss the publications on LPA and GBM, ovarian, breast, and colorectal cancers over the last 6 years.

Regarding colon cancer, it has been shown that LPA and WNT induce proliferation of these cells by activating β-catenin in a synergistic fashion and that Krüppel-like factor 5 (KLF5) acts as a cofactor in this process, facilitating the interaction between β-catenin and TCF4.^190^ Moreover, LPA has been linked to the induction of DNA synthesis and migration of colorectal cancer cells, probably in an EGFR-independent manner.^191^ Still, LPA has been correlated with S1P in colorectal cancer, since there were higher mRNA levels of LPAR2 and sphingosine kinase (SphK1), an oncogenic kinase that produces S1P, in tumor tissues when compared to normal tissue.^192^ Furthermore, it has been demonstrated that LPAR4 and LPAR6 negatively regulate the proliferation and migration induced by LPA on colon cancer cells.^193^ LPA also was shown to induce the proliferation of colon cancer cells via RhoA-ROCK and STAT-3 pathways associated with cell cycle progression.^194^

In breast cancer, it was shown that the high levels of ATX, which culminates in increased LPA production, are mainly originated from adipose-derived stem cells and adipocytes.^195^ An interesting recent study showed a direct mechanism involving LPA that links obesity and breast cancer progression, where it was revealed that in a breast cancer model under chronic diet-induced obesity LPA/PKD-1-CD36 signaling stimulated microvascular remodeling.^196^ LPA is also implicated in breast cancer metastasis to bone, as it induces the expression of IL-8, IL-11, and osteolytic cytokines in breast cancer cells.^197^ In another work, the proliferation and migration promoted by LPA and EGF in breast cancer cells were inhibited not only by n-3 fatty acid eicosapentaenoic acid but also by free fatty acid receptor (FFAR) agonizts, lighting up a possible anticancer role for FFAR.^198^ Concerning breast cancer cells motility, it was shown that LPA induces two different signaling pathways: Rho/ROCK, inducing a slow, directional, coherent, and consistent movement; and G_αi/o_ and G_α11/q_ dependent, promoting a lessen directionality and increase independent movement, favoring cell dispersal; so the balance between them defines the migratory reaction of cells, all depending on the cellular or microenvironmental context.^199^ Still, LPA was proven to induce breast cancer invasion via YB-1/EZH2/amphiregulin signaling axis^200^ and RhoA/ROCK/MMPs signaling pathway;^201^ further, curcumin was unprecedentedly shown to inhibit this effect by attenuating the RhoA/ROCK/MMPs pathway.^201^ In contrast, it has recently been reported that LPAR6 is downregulated in breast cancer, relating it to poor prognosis, which might implicate LPAR6 as a tumor suppressor gene in these cancers.^202^

LPA has a tremendously described influence on ovarian cancer. It acts on the EMT, glucose metabolism, cell migration, among other roles; and also functions as a biomarker. Recently, it has been shown that LPA can induce EMT through the activation of Wnt/β-catenin signaling^183^ as well as via G_αi2_, Src, and HIF1α signaling nexus,^183^ and by repressing SIRT1.^186^ LPA also stimulates glycolytic metabolism through induction of HK2, promoting tumor cell proliferation^203^ and pseudohypoxic response, inducing aerobic glycolysis and has a pro-tumor role.^204^ It is also suggested that this glycolytic shift promoted by LPA is a priming event in the transition from normal to cancer-associated fibroblasts.^205^ LPA has been described as a biomarker of ovarian cancer regarding its high levels in plasma^206,207^ and the expression of its receptor in tissue,^208^ leading to the investigation of a biosensor to detect LPA in the serum of patients^209^ and even to the development of a device for fast detection of plasma LPA.^210^ Nevertheless, this plasma detection should be addressed carefully since it has been reported that sample processing and analysis could interfere in LPA levels.^211^ Still, it has been reported that LPA might also become a biomarker in vaginal fluid, but only for endometrioid ovarian cancer, being even more sensitive than the classical marker CA125.^212^ LPA promotes migration of ovarian cancer cells through different mechanisms/signaling pathways: RhoA/ROCK and Rac1/PAK,^213^ CXCL12‑CXCR4,^214^ uPA/uPAR,^215^ G_αi2_/Src/β-pix/Rac,^216^ FOXM1,^217^ LPAR1,^218^ NHERF1/cpERMs,^219^ ETS-1,^186^ LPAR1/LPAR2/ERM,^220^ LPAR2/ERK, LPAR3/ERK, and LPAR6/AKT.^221^ LPA has also been implicated in the maintenance of cancer stem cell characteristics through ATX–LPA–LPAR1–AKT1 signaling axis in an autocrine loop.^222^ Moreover, LPA treatment leads to morphological change and alterations in the relative protein-to-lipid ratio on the surface of ovarian cancer cells.^223^ Interestingly, it has been shown that LPA in ascites is produced via PLA_2_ and ATX secreted by tumor-associated macrophages, where the 20:4 LPA species seems to have an important role in tumor recurrence.^224^ In fact, regarding the use of LPA signaling blockade as a possible therapy, it has been found that it increases cell death in response to chemotherapeutic agents.^225^

Lastly, GBM is the most common and deadliest primary malignant tumor of the CNS,^226,227^ and it has been a main focus of interest of our group. In this LPA context, ATX is highly upregulated and favors GBM migration/invasion.^109,228–230^ In GBM, LPAR1 signaling was shown to be important for tumor cell migration/invasiveness.^109,229,231,232^ Also, LPA signaling is implicated in tumor recurrence, and cancer stem cells have higher expression of LPAR1.^231,233^ Finally, our group recently reported that high LPAR1 and ATX correlates with glioma aggressiveness and patient prognosis.^234^ Also, we described that microglial cells, the major source of LPA in the CNS,^230^ secrete LPA in the TME, which induces GBM cell migration and proliferation via LPAR1.^234^

All these findings show that LPA signaling is an important pathway in carcinogenesis, tumor progression and establishment of the TME. In this sense, components of the ATX–LPA–LPARs axis can be important biomarkers or therapeutic targets for the treatment of different cancers,^231,235^ some of which will be reviewed in the next topic.

## LPA signaling as a therapeutic target

Since the discovery that LPAR1 is overexpressed in samples from patients with idiopathic pulmonary fibrosis (IPF) and kidney fibrosis, several groups tried to target LPARs or ATX with small molecules to inhibit fibrosis. IFP is a chronic disease characterized by excessive collagen deposition in the lungs. ATX is known to be involved in the pathogenesis of IPF by increasing collagen production and migration of inflammatory cells to the lungs.^236^ In addition, LPAR1 knockout mice show a reduction in both renal and pulmonary fibrosis.^237–239^

Amira Pharmaceuticals developed the first highly selective LPAR1 inhibitors, AM966 and AM095. In vivo, oral administration of AM966 and AM095 reduced lung fibrosis, lung collagen deposition, vascular leakage, and inflammation.^240,241^ Moreover, AM095 inhibited kidney fibrosis induced by unilateral ureter obstruction and dermal fibrosis induced by bleomycin in murine models.^238,240^ Both inhibitors antagonize LPA-induced Ca^+^ release selectively in LPAR1 expressing cells by blocking the binding of guanosine 5′-O-[gamma-thio]triphosphate (GTPγS) to membranes induced by ligation of LPA to LPAR1.^238,240,241^

Other inhibitors of LPAR1 have already been tested in patients with IPF: BMS-986020 was tested in a phase 2 trial between April 2013 and February 2016. Patients had a minor improvement in disease control when compared to the placebo group, as stabilization of forced vital capacity (FVC) and improvement of lung fibrosis and inflammation biomarkers (C3M and adiponectin). Nevertheless, 80% of the patients in the treated group experienced hepatic adverse effects, and the trial was early terminated due to 3 cases of BMS-986020-related cholecystitis.^242^

Another phase 2 clinical trial was conducted with a small molecule antagonist of LPAR1 (SAR100842) for patients with diffuse cutaneous SSc. Overall, SAR100842 was safe and 80% of the patients in the treated group reported only minor adverse effects, compared to 71% in the placebo group.^243^

Ono Pharmaceuticals developed a selective ATX inhibitor, ONO-8430506. It is a tetrahydrocarboline derivative with a half-maximal inhibitory concentration (IC_50_) of approximately 10 nM.^244^ This compound was able to block the formation of LPA in the plasma of rats even after 24 h of treatment. The effect of ONO-8430506 endures for a longer period when compared to other ATX inhibitors.^244^ Since LPA induces the contraction of the urethra, the authors tested if ONO-8430506 could also inhibit this effect. The compound was able to decrease intraurethral pressure similarly to clinically used tamsulosin, an α1-adrenoceptor antagonist, without changing mean blood pressure.^244^ This inhibitor was also proven to have antitumor effects in preclinical metastatic breast and thyroid cancer models,^188,245,246^ showing the versatile potential of ATX inhibitors for the treatment of human diseases.

Recently, GLPG1690, a first in class drug that selectively inhibits ATX with an IC_50_ of approximately 100 nM^247^ was tested for the treatment of IPF. In a mouse model of bleomycin-induced pulmonary fibrosis, GLPG1690 showed efficacy in reducing extracellular matrix deposition in the lungs and LPA levels in plasma. GLPG1690 showed superior results compared to pirfenidone and was similar to nintedanib, a tyrosine kinase inhibitor, in reducing the Ashcroft fibrotic score and collagen levels.^247^ GLPG1690 was already tested in humans in order to test the safety, pharmacokinetics and pharmacodynamics of its doses,^248^ and was well tolerated in a Phase 2a study (FLORA study) involving 23 patients with IPF. Treated patients had reduced plasma LPA C18:2 levels and showed improvement of disease control, such as stabilized FVC after 12 weeks of treatment.^249^ Maher et al.^249^ started in November 2018 two identically designed phase 3 studies (Isabela I and II) to test GLPG1690 in 750 patients with IPF (see Table 3).

**Table 3:** Clinical trials using LPA signaling as a therapeutic target for different pathologies^a^

Study	ClinicalTrials.gov Identifier	Sponsor	Disease	Phase	Drug inhibitor
Safety and efficacy of a LPA receptor antagonist in idiopathic pulmonary fibrosis	NCT01766817	Bristol-Myers Squibb	Idiopathic pulmonary fibrosis	2	LPAR1 inhibitor drug: BMS-986020
Safety, tolerability, pharmacokinetics, and pharmacodynamics of BBT-877 in healthy subjects	NCT03830125	Bridge Biotherapeutics, Inc.	Idiopathic pulmonary fibrosis	1	ATX inhibitor
Phase 2a, open-label study of two doses of GLPG1837 in subjects with cystic fibrosis and the S1251N mutation	NCT02690519	Galapagos NV	Cystic fibrosis	2	ATX inhibitor
Phase 2a, open-label study of multiple doses of GLPG1837 in subjects with cystic fibrosis and the G551D mutation	NCT02707562	Galapagos NV	Cystic fibrosis	2	ATX inhibitor
Evaluation of the pharmacokinetics, safety and tolerability of a single dose of GLPG3067 administered as solid formulation in male subjects with cystic fibrosis	NCT03589313	Galapagos NV	Cystic fibrosis	1	ATX inhibitor
Evaluation of the pharmacokinetics, safety and tolerability of a single dose of GLPG2737 administered as oral suspension in male subjects with cystic fibrosis	NCT03450720	Galapagos NV	Cystic fibrosis	1	ATX inhibitor
Phase 2a, randomized, double-blinded placebo-controlled study to evaluate GLPG2737 in orkambi-treated subjects with cystic fibrosis homozygous for the F508del mutation	NCT03474042	Galapagos NV	Cystic fibrosis	2	ATX inhibitor
Phase 2a, randomized, double-blinded, placebo-controlled study to evaluate GLPG2222 in ivacaftor-treated subjects with cystic fibrosis harboring one F508del CFTR mutation and a second gating (Class III) mutation	NCT03045523	Galapagos NV	Cystic fibrosis	2	ATX inhibitor
Assessment of safety, tolerability, pharmacokinetics and pharmacodynamics of multiple oral doses of the combination of GLPG2451 and GLPG2222, with or without GLPG2737, in adult subjects with cystic fibrosis	NCT03540524	Galapagos NV	Cystic fibrosis	1	ATX inhibitor
Phase 2a, randomized, double-blind, placebo-controlled study to evaluate multiple doses of GLPG2222 in subjects with cystic fibrosis who are homozygous for the F508del mutation	NCT03119649	Galapagos NV	Cystic fibrosis	2	ATX inhibitor
Phase 2 randomized, double-blinded, placebo-controlled, 26-week study to evaluate the efficacy, safety and tolerability of GLPG1205 in subjects with idiopathic pulmonary fibrosis	NCT03725852	Galapagos NV	Idiopathic pulmonary fibrosis	2	ATX inhibitor
Phase 3, randomized, double-blinded, parallel-group, placebo-controlled, multicenter study to evaluate the efficacy and safety of two doses of GLPG1690 in addition to local standard of care for minimum 52 weeks in subjects with idiopathic pulmonary fibrosis	NCT03733444	Galapagos NV	Idiopathic pulmonary fibrosis	3	ATX inhibitor
Phase 3, randomized, double-blinded, parallel-group, placebo-controlled multicenter study to evaluate the efficacy and safety of two doses of GLPG1690 in addition to local standard of care for minimum 52 weeks in subjects with idiopathic pulmonary fibrosis	NCT03711162	Galapagos NV	Idiopathic pulmonary fibrosis	3	ATX inhibitor
Randomized, double-blind, parallel group, placebo-controlled, multicenter, exploratory phase 2a study to assess safety, tolerability, pharmacokinetic and pharmacodynamic properties of GLPG1690 administered for 12 weeks in subjects with idiopathic pulmonary fibrosis (IPF)	NCT02738801	Galapagos NV	Idiopathic pulmonary fibrosis	2	ATX inhibitor
Double-blinded, randomized, 8-week placebo-controlled, and 16-week open-label extension study investigating the safety, pharmacokinetics and pharmacodynamics of SAR100842 given orally to patients with diffuse cutaneous systemic sclerosis	NCT01651143	Sanofi	Systemic sclerosis	2	LPAR1 inhibitor SAR100842

^a^Data extracted from www.clinicaltrials.gov

Finally, a new selective ATX inhibitor, BI-2545, was developed based on a previously published inhibitor by Pfizer (PF-8380). This improved inhibitor was able to reduce plasma LPA levels by 90% and with a higher in vivo potency compared to PF-8380.^250^

In the last 10 years, researchers have searched for multi-target drugs in order to treat complex diseases. IFP is also correlated to lung cancer which occurs as a late complication of the fibrotic tissue. EGFR inhibitors are effective in the treatment of non-small cell lung cancer (NSCLC). Thus, the discovery of a dual inhibitor for EGFR and ATX could be of great importance for treating not only IFP but also its late complications as NSCLC. Tetrahydropyrido[4,3-d]pyrimidine was found, by in vitro cytotoxic and enzymatic assays, to inhibit both ATX and EGFR with anticancer and antifibrotic activity superior to gefitinib, an EGFR inhibitor clinically used for the treatment of lung, breast and other cancers with overactive EGFR signaling. Tetrahydropyrido[4,3-d]pyrimidine also inhibits the expression of fibrotic markers, as TGF-β and tumor necrosis factor alfa (TNF-α).^251^

LPA and ATX play key roles in the progression of various cancers since one of the major hallmarks of cancer is cell migration. GBM is the most malignant brain tumor with high infiltration capacity in the cerebral parenchyma and both ATX and LPAR1 are highly expressed in several types of brain cancers. Motility of GBM cells seems to depend on ATX and LPAR1. Treatment of GBM cell line with LPAR1 antagonist, Ki16425, inhibits dramatically migration of these cells in vitro.^110,234^ Both α-bromomethylene phosphonate, a dual LPA receptor inhibitor/ATX inhibitor, and PF-8380, an ATX inhibitor, delayed in 20 days the growth of GL261 GBM cells injected in the hind limbs of nude mice.^252,253^

Since LPA induces secretion of proinflammatory cytokines and excessive scar formation, inhibition of the LPA pathway may also be an efficient strategy to improve brain and spinal cord damage. A mouse monoclonal antibody against LPA, called B3, was able to abolish the inhibitory effect of LPA on neurosphere formation and neuronal differentiation of human embryonic stem cells in vitro. Furthermore, B3 was also able to decrease astrogliogenesis, activation of microglia and neuronal apoptosis in a mouse model of spinal cord injury, reducing ERK phosphorylation and Rho-GTP levels.^254^ LPA is also highly secreted in TBI, both in humans and in mouse models as mentioned above. This antibody later renamed LpathomabTM reduced the size of hematoma and lesion 7 days after TBI, and also improved mice’ motor functions.^162^ In addition, LpathomabTM decreased IL-6 expression, an inflammatory cytokine correlated with poor outcome after TBI. This led the authors to conclude that anti-LPA antibody therapy could have neuroprotective effects following brain injury.^162^

In addition, it has been suggested that LPA and especially variations in the levels of LPA within different body fluids (e.g., serum, plasma, saliva, seminal plasma, and follicular fluid, among others) might be used as potential biomarkers to the determine the diagnosis and severity of diseases.^255,256^ In fact, LPA has been considered a biomarker in the diagnosis of ovarian cancer.^257^ Moreover, higher of LPA levels have been detected in the plasma of patients with renal failure^258^ as well as in the serum multiple myeloma patients.^259^ The production of LPA in the lung of asthmatic patients as well as the value of polyunsaturated LPA molecular species obtained from the bronchoalveolar lavage fluid have been also discussed as possible biomarkers not only to stablish the diagnosis but also to determine the severity of the disease.^260^

## Conclusions

In summary, the current literature points to LPA regulating diverse important biological processes, and the cell response depends on the environment, cell type and developmental stage. Targeting LPA receptors, particularly using antagonist and agonist molecules or knockout mice, research groups have demonstrated several roles for LPARs in many disease models and also during embryo development.^261^ There is strong evidence regarding a chief role of LPA in cancer and neurodegenerative diseases as we explored here. There is no doubt that LPA and its receptors, LPAR1 and LPAR3, are crucial not only for the establishment but also for the chronification of neuropathic pain.^122^ Considering the huge literature supporting the role of LPA in so many diseases, it is important to explore new pharmacological agents capable of blocking the actions of different species of LPA without producing significant side effects.
